# Multimodal exploration of the intracranial aneurysm wall

**DOI:** 10.1007/s11548-023-02850-0

**Published:** 2023-03-06

**Authors:** Annika Niemann, Riikka Tulamo, Eliisa Netti, Bernhard Preim, Philipp Berg, Juan Cebral, Anne Robertson, Sylvia Saalfeld

**Affiliations:** 1 Department of Simulation and Graphics, Otto-von-Guericke University, Magdeburg, Germany; 2 STIMULATE Research Campus, Magdeburg, Germany; 3 Faculty of Medicine, University of Helsinki, Helsinki, Finland; 4 Department of Medical Engineering, Otto-von-Guericke University Magdeburg, Magdeburg, Germany; 5 Computational Hemodynamics Lab, Georg Mason University, Fairfax, USA; 6 Department of Mechanical Engineering and Materials Science, University of Pittsburgh, Pittsburgh, USA

**Keywords:** Intracranial aneurysm, Multimodal visualization, Histology

## Abstract

**Purpose:**

Intracranial aneurysms (IAs) are pathological changes of the intracranial vessel wall, although clinical image data can only show the vessel lumen. Histology can provide wall information but is typically restricted to ex vivo 2D slices where the shape of the tissue is altered.

**Methods:**

We developed a visual exploration pipeline for a comprehensive view of an IA. We extract multimodal information (like stain classification and segmentation of histologic images) and combine them via 2D to 3D mapping and virtual inflation of deformed tissue. Histological data, including four stains, micro-CT data and segmented calcifications as well as hemodynamic information like wall shear stress (WSS), are combined with the 3D model of the resected aneurysm.

**Results:**

Calcifications were mostly present in the tissue part with increased WSS. In the 3D model, an area of increased wall thickness was identified and correlated to histology, where the Oil red O (ORO) stained images showed a lipid accumulation and the alpha-smooth muscle actin (aSMA) stained images showed a slight loss of muscle cells.

**Conclusion:**

Our visual exploration pipeline combines multimodal information about the aneurysm wall to improve the understanding of wall changes and IA development. The user can identify regions and correlate how hemodynamic forces, e.g. WSS, are reflected by histological structures of the vessel wall, wall thickness and calcifications.

## Introduction

Intracranial aneurysms (IA) are pathological vessel wall dilations that could rupture with fatal consequences for the patient. Asymptomatic IAs might not rupture and treatment would induce unnecessary risks for the patient [1]. Therefore, a therapeutic decision must be made carefully, which is complicated as the hemodynamic and biomechanic mechanisms leading to aneurysm formation and aneurysm rupture are not fully understood yet. IA rupture risk assessment based on clinically available data, such as medical images as well as morphological and hemodynamic analysis, is an active research area. However, imaging of the vessel wall and the adjacent flow fields, where the pathology is manifested, is missing.

The combined analysis of histology and hemodynamics can reveal important insights into the wall composition and its transformation from healthy to pathological tissue. However, this can only be applied to resected tissue. Due to changes during the fixation and preparation process, in combination with the original shape and other image modalities is cumbersome and a fully automated solution is not yet available.

In this study, we provide a visual exploration approach for histologic image data with four different stains of a resected IA dome, combined with micro-CT and hemodynamic parameters extracted from the in vivo 3D IA model. Due to the differences in stainings, no landmarks inside the tissue could be used for registration to micro-CT. A further challenge for registration was the non-compact tissue shape, yielding arbitrary deformations. Additionally, large gaps between the images may occur with drastic differences between consecutive slices.

As current imaging modalities are unable to image the IA wall using non-invasive techniques, there is no ground truth available before collection. At the moment, clinical researchers have to mentally combine histologic and pre-operative images.

To overcome this unsatisfactory situation, we make the following contributions:

- A user interface to guide the alignment of histologic images with micro-CT images with respect to technical limitations of histologic sectioning.
- A virtual inflation to restore the shape of the tissue collected during surgery based on preoperative imaging.
- A mapping approach to combine information of images with insufficient similarity for registration.
- A pipeline for multimodal visual exploration of the IA wall.

## Related work

In addition to the introduction section, related work is summarized in the following. Specifically, the acquisition of relevant hemodynamic parameters, the subsequent multi-modal wall analysis, as well as studies regarding tissue deformation and histology, are addressed.

### Near-wall hemodynamics.

Since hemodynamic forces on the lumen of IAs are very difficult to acquire, image-based blood flow simulations demonstrate an important technique to allow a quantitative assessment of the individual pathological state. Several sophisticated review articles demonstrated already the importance of the central hemodynamic parameter wall shear stress (WSS), as a result of tangential shear forces, concerning aneurysm initiation, remodeling and rupture, respectively [2–5]. Furthermore, numerous international research challenges focused on relevant modeling aspects such as reliable reconstruction and segmentation [6,7], profound simulation [8–10] and independent validation [11]. Therefore, individualized blood flow simulations in combination with wall-related imaging can contribute valuable information w.r.t. the presented multimodal visual exploration pipeline. Particularly, the interaction between wall thickness and WSS is subject to research, reportedly with thin vessel walls possibly correlating to very low or very high WSS [12].

### Multimodal aneurysm wall analysis.

While most studies focus on a single aspect, like morphological or the latter mentioned hemodynamic parameters, the following approaches combine multiple modalities. Jiang et al. [13] analyzed 28 middle cerebral artery IAs. They performed hemodynamic simulations using models based on preoperative CT angiography (CTA). The simulation results were combined with information about thin-wall regions manually identified by intra-operative microscopy. Thin-wall regions correlated with higher pressure and lower WSS. Cebral et al. [14] analyzed 65 aneurysms from preoperative imaging. Based on surgical videos, five different wall types (atherosclerotic wall, hyperplastic wall, thin wall, rupture site, and normal) were identified and marked on the extracted 3D models. In the videos, 28.9%±25.6% of the IAs were not visible, but the remaining parts were compared with the hemodynamic simulation results. Slow flow was associated with an atherosclerotic and hyperplastic wall, while high flow was associated with wall thinning.

### Tissue deformations.

The post-mortem ex-vivo analysis of IAs is challenging, as the shapes of the aneurysms are altered during the extraction and fixation process. The main difficulty is the collapsing of the vessel and aneurysm due to the loss of blood flow inside. The virtual inflation approach based on optical coherence tomographic and histologic data of ex-vivo intracranial vessels projects the inner vessel wall on a circle and transfers the wall tissue accordingly [15]. Athanasiou et al. [16] proposed virtual inflation for histology and micro-CT of vessels. Their inflation first requires manual registration of the images to intravascular ultrasound. The inflation is then based on the differences in the vessel contours. As the tissue is deformed during collection, we perform a virtual inflation based on preoperative imaging.

### Histology and 3D information.

In the past, several studies have combined micro-CT and histology, with the latter used to verify diagnoses based on other image modalities (without registration) or manually matched to the micro-CT data. Jessen et al. [17] used micro-CT images and histologic images to assess the response to elastase-induced aneurysm treatment in rabbits with coils without carrying out a registration. During the analysis of the histologic images, the experts used the micro-CT images as a reference for the original coil orientation. Griffiths et al. [18] correlated diffraction micro-CT images with histopathological images of breast tissue sections. Falk et al. [19] presented an interactive visualization of 3D histopathology. They used over a hundred consecutive images to create a volumetric dataset from histologic images. In previous work, we extracted a 3D IA model based on adjacent histologic H&E-stained slices, but no in-vivo model of the IA was available since it was explanted post-mortem [20].

Neither approach can be used for our data due to the variation of different histologic stainings. Although they reveal various aspects of the tissue, they hinder a landmark- or feature-based registration.

## Materials and methods

**Fig. 1:**
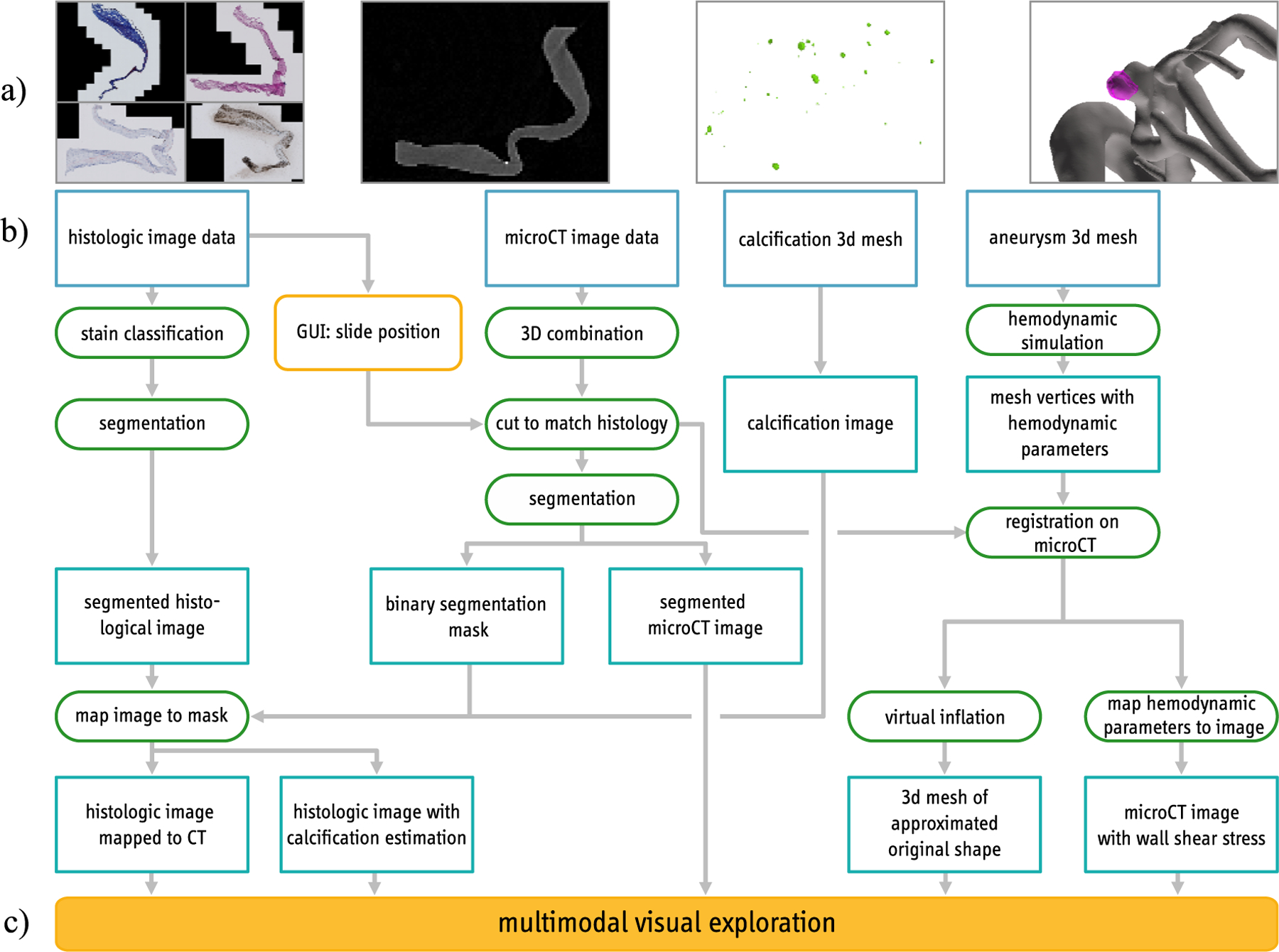
Pipeline overview with **a** histologic and micro-CT data, calcification segmentation and 3D IA surface model (gray) and 3D resected dome surface model (pink), **b** the steps to combine this data and **c** the resulting visual exploration

In the following, the medical image data and our pipeline are described (see Fig. 1). All programming steps were carried out in MATLAB (Version R2020a, The MathWorks Inc., Natick, Massachusetts, U.S.). The presented pipeline combines histologic image data, micro-CT image data, calcification information from micro-CT image data and a preoperative aneurysm mesh. The data is combined in a multimodal visual exploration tool.

### Image data

The multimodal data was acquired from a patient with an IA at the middle cerebral artery. Pre-operative 3D angiography data was used to extract a 3D surface model of the aneurysm and its parent vessel. A threshold-based approach was used to segment the vessels including the IA from the image data and vessels are truncated perpendicularly to their axes [21]. Image data from clinical routine with an isotropic voxel resolution of approximately 0.3mm are sufficient for surface mesh extraction.

**Fig. 2:**
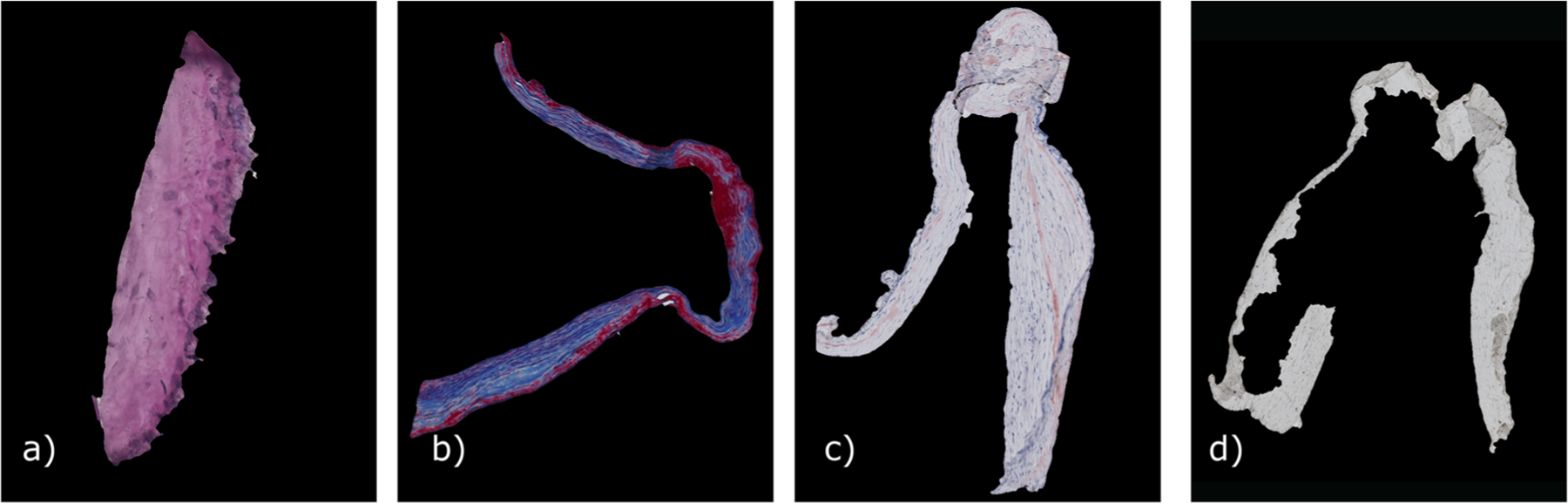
Segmentation of different histologic stainings, **a** H&E, **b** MT, **c** Oro and **d** aSMA

The patient underwent microsurgical clipping, where a metal clip closes the aneurysm head and separates it from the parent vessel. During surgical intervention, the aneurysm dome was resected and scanned afterwards via micro-CT yielding a stack of micro-CT image data [22]. The high-resolution Skyscan 1272, 11Mp micro-CT scanner (Bruker Corporation) was used with the following settings: camera pixel size of 7.4 μm, image pixel size of 10 μm, frame averaging of 10 with a rotation step size of 0.4°, scanned 180° around the vertical axis. The reconstructed 3D image data exhibit 10 μm isotropic resolution. From this dataset, calcification masks were generated [22]. Next, histologic images based on the fixated, stained and sliced aneurysm dome were scanned. Four histologic stains were used: (1) Hematoxylin and eosin (H&E), (2) alpha-smooth muscle actin (aSMA), (3) Oil Red o (ORO) and (4) Masson trichrome (MT), see Fig. 2. The available data are shown in Fig. 1a.

### Requirements

Based on the related studies, the IA wall exploration has to fulfill several requirements.

1. *Account for different stainings:* Each stain only shows specific characteristics of the tissue. To obtain a comprehensive understanding of the aneurysm wall, multiple stains must be combined.
2. *Account for large gaps and missing data:* Due to the unique image acquisition of histologic images, there might be large gaps between consecutive slices. Slides can be missing.
3. *Account for tissue deformation:* While most of the data (micro-CT, histology) is based on tissue collected during surgery, the hemodynamic parameters are based on a pre-operative IA model. During the collection and processing of the tissue sample, the shape of the tissue is alternated.
4. *Combine 2D and 3D data:* The 2D histologic images have to be combined with 3D data.

### Methods

#### Histologic image processing

The IA tissue was segmented in the histologic images. The different staining methods resulted in different colors and saturations. H&E and MT were darker and could be easily distinguished from the light background in contrast to ORO and aSMA stainings. The size of the images varied, with large image sizes (> 10,000 × 10,000 pixels) being common. The program automatically determines the stain with a neural net based on the GoogleNet architecture [23] trained on 70 RGB images with a size of 500 × 500 pixels.

For aSMA, H&E and ORO-stained images, two thresholds were determined using Otsu’s method [24]. One threshold separated the object from the black padding of the scanning process, and the other roughly segmented the tissue from the slide background yielding a binary segmentation mask. As the slides can contain some dissected tissues or other impurities, the mask may falsely contain small objects that were removed. The final segmentation was refined with geodesic active contours [25] using the masks as an initial state. These steps required 2–3 min.

#### Co-alignment of histology with micro-CT images

Co-registration of micro-CT image data with histologic slices is challenging due to shape alternation of the tissue sections (tissue sections may slip away or stainings could fail), folding artifacts, large gaps between slices and the usage of four different stainings.

**Fig. 3:**
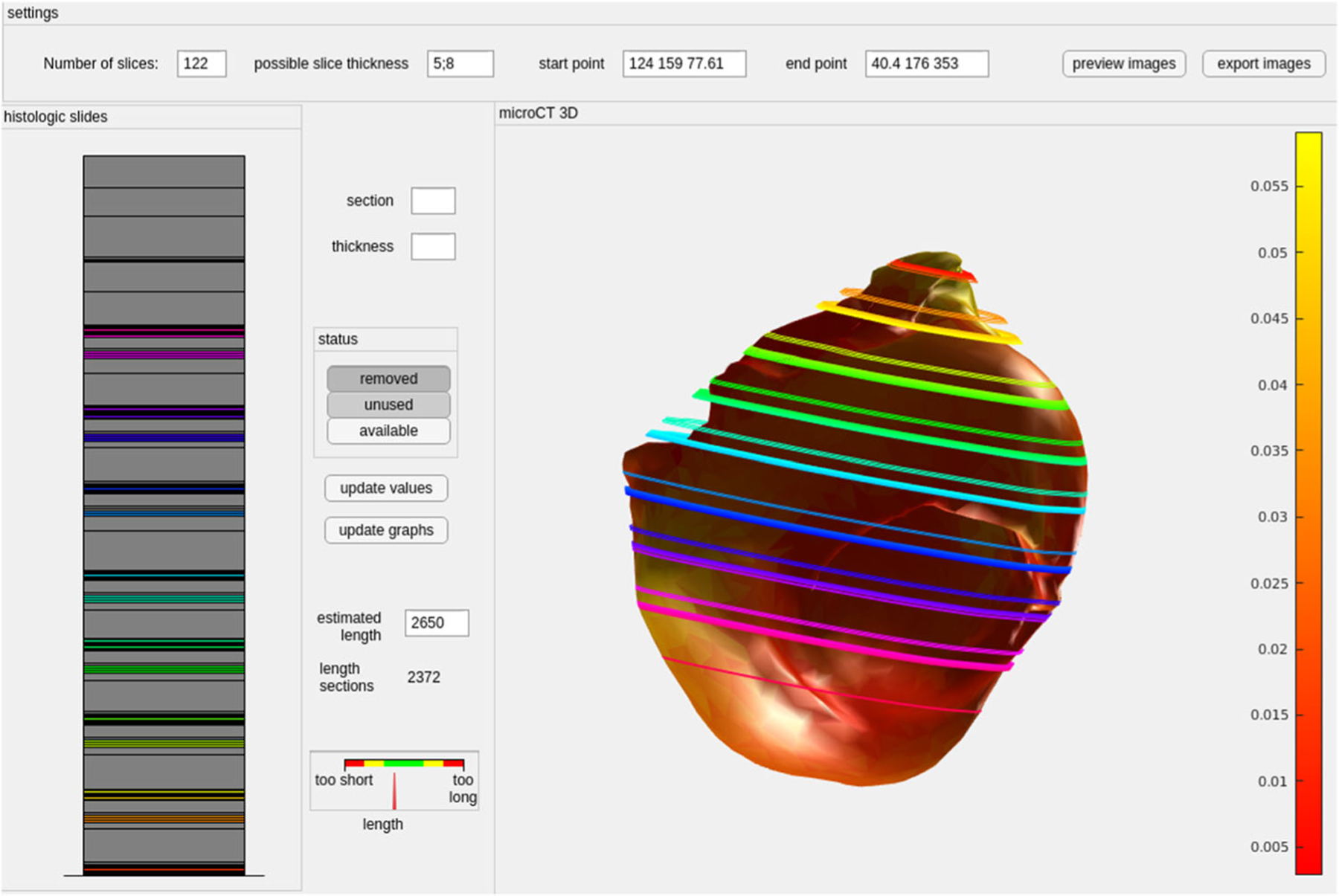
User interface for alignment of the histologic slides. The sections (colored: available, gray: removed tissue or unavailable slides) are shown left. On the right, a 3D model extracted from micro-CT is shown together with the estimated slide positions. The mesh is colored with the mesh thickness (color bar)

To overcome these limitations, we used a semi-automatic approach, where a 3D surface model is generated from the micro-CT data. Next, histologic images were aligned along the micro-CT 3D model based on the histologic preparation protocol, see Fig. 3. The number of slices (including placeholder slices for removed sections) and slice thickness values were set. These are shown on the left side as a stack. Based on the provided information, the micro-CT 3D model is shown with the estimated slide positions. The user can set the estimated length of the tissue sample, for example, based on measures of the CT model, and compare this to the sum of the histologic slides. Due to the user input, no automatic assessment of the required time was possible but usually, these steps required approximately 3–4 min.

#### Virtual inflation of the aneurysm sac

**Fig. 4:**
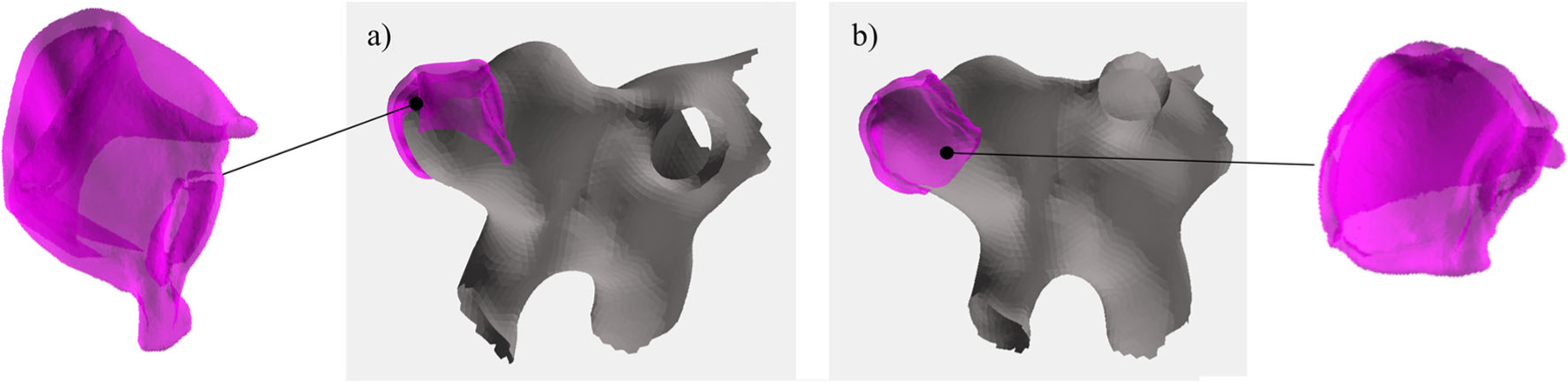
In (**a**), the 3D aneurysm mesh (grey) and resected, deflated dome mesh (pink) is shown. In (**b**), the virtual inflation was applied to the dome surface mesh

The resected aneurysm dome acquired with micro-CT data is deflated due to explantation and does not correspond well to the original in-vivo shape, see Fig. 4. We therefore virtually inflated the resected dome for a future co-alignment with the other modalities. First, the resected dome mesh vertices are split into inner and outer points. The inner points of the bowl-shaped dome sample are the points that build the outline of the aneurysm lumen and should follow the shape of the pre-operative aneurysm mesh (recall in “Image data” section). A rough split can be achieved by calculating which vertices are visible from the center *C* of the resected dome mesh, where *C* is extracted as the mean of the points of the surface mesh. The line *l*_*i*_ is defined between surface model vertex *v*_*i*_ and *C*:

(1) $$l_{i}=v_{i}-C$$

Let *M* be the set of points inside the mesh. For each vertex *i* the initial label (0 for inner points, 1 for outer points) is assigned by the function f:

(2) $$f(i)=\{\begin{array}{ll} 0 & \forall x\in l_{i}∄x\in M\cap l_{i}\\ 1 & \text{else } \end{array}$$

Afterwards, this classification is refined by assigning each vertex *v*_*i*_ the label of the majority of its neighbors, i.e., all vertices that share an edge with *v*_*i*_.

Next, the inner vertices are iteratively moved to the center of the closest aneurysm mesh vertices. The outer vertices are moved accordingly to the average movement of the closest inner points to maintain the local wall thickness.

For the presented approach, the 3D surface mesh, extracted from preoperative patient data, served the as ground truth for the amount of the virtual inflation. This step took less than a minute. For more information about the virtual inflation approach, please refer to [15].

#### Hemodynamic simulation for wall shear stress extraction

The preoperative 3D IA model was used for obtaining the desired near-wall hemodynamic parameters. The corresponding simulation was carried out in STAR-CCM+ 2020.1 (Siemens PLM Software Inc., Plano, TX, USA). Blood was modeled as an incompressible and Newtonian fluid with a density of 1055 $\frac{\text{kg}}{{\text{m}}^{3}}$ and dynamic viscosity of 0.004 Pas. Boundary conditions of the domain were modeled as follows: Time-dependent flow waveform from a healthy volunteer, rigid vessel walls with no-slip condition, and zero-pressure assumption at the outlets [26]. Based on the hemodynamic simulation, a highly-resolved WSS field is extracted. The time-dependent simulation representing one cardiac cycle required approximately 4h.

#### Multimodal exploration

**Fig. 5:**
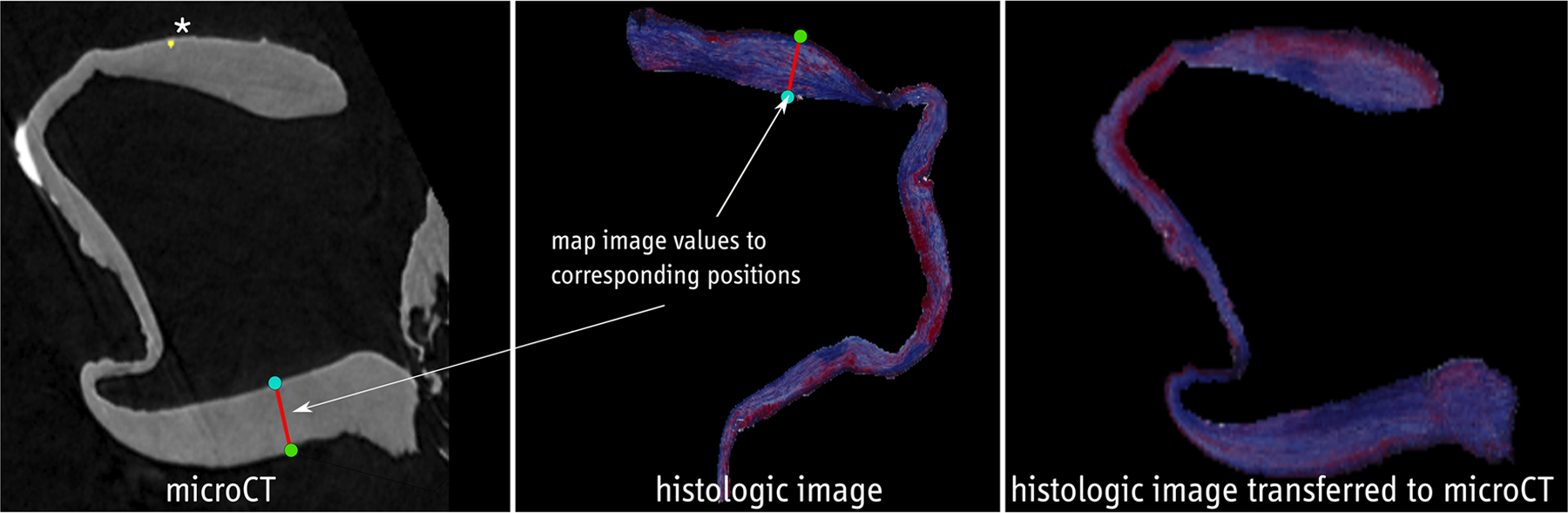
Transferring histologic image information to the shape of micro-CT image with calcification (*); cyan: inner contour point, green: outer contour point, red line: image values between inner and outer point are mapped to the corresponding position in the micro-CT image

After the preprocessing, for each histologic image, a corresponding position in the micro-CT data with segmented calcifications is available. Next, co-alignment is carried out to provide more detailed information, see Fig. 5. The contour of the histologic and the micro-CT images are each split up into inner and outer contour points. Here, the center of the contour is calculated. For each contour point, intersections of the line between the center and the contour point and the polygon described by the contour are determined. If no intersections besides the contour point itself are found, the point belongs to the inner contour, otherwise, it belongs to the outer contour.

The information from the histologic images is then transferred to the segmentation mask of the micro-CT image. Every image is split up into 5000 overlapping, small sections. Each of these sections includes one inner contour point and 50 outer contour points. The first and last sections contain the ends of the u-shaped tissue with minimum distance between the outer and inner points. The image values from the first section of the histologic image are mapped to the first section of the micro-CT image and so on, see Fig. 5. As the sections overlap, a pixel of the micro-CT image can get several values from the histologic image. In that case, the average is used. To avoid blurring, the number of times a pixel value can be updated is limited to 5.

A section *a* of the histologic image consists of the inner contour point *I*_*a*_ and the outer contour points *O*_*ai*_. It is mapped to the corresponding section *b* in the micro-CT image consisting of the inner contour point *I*_*b*_ and the outer contour points *O*_*bi*_. The function *f*_*i*_*(x)* describes the image values along the line segment *a*_*i*_ between *O*_*ai*_ and *I*_*a*_. The function *g*_*i*_*(x)* describes the image values along the corresponding line segment *b*_*i*_.

(3) $$g_{i}(x)=\frac{\left\|b_{i}\right\|}{\left\|a_{i}\right\|}*f_{i}(x)$$

The value at the pixel p in the micro-CT image is calculated using several functions *g*_*n*_ from different, overlapping sections.


(4) $$v(p)=\overset{5}{\underset{n=1}{\sum }}g_{n}(p)$$

For a mapping of numerically acquired hemodynamic information (e.g., WSS), the values are assigned to each vertex of the 3D IA surface model. The 3D surface of the resected IA dome is co-registered to the 3D surface extracted from the micro-CT data via the iterative closest point algorithm [27]. The transformation matrix from this co-registration is then applied to the 3D IA surface model as well. Finally, each voxel of the micro-CT is assigned the parameter values of the closest aneurysm mesh vertex.

Similar to the micro-CT data, the information of the histologic images is mapped to the inflated 2D shapes as well. Since the interactive exploration relies on preprocessed data, the co-alignment and update of the GUI require only a few seconds.

## Results

Our prototype allows for the visual exploration of histologic slices (cut parallel and with a given thickness) arranged in a 3D model. Furthermore, near-wall hemodynamics can be assessed and associated with histologic anomalies.

### Evaluation of the wall exploration requirements

We integrated an automatic stain classification based on a neural net. We trained with 70 images, tested on 8 (two of each staining), and achieved an accuracy of 100%. The inclusion of stain classification followed by stain-specific segmentation fulfills requirement 1.

The graphical user interface allows the mapping of the available slices to a 3D model. Large gaps between slices (req. 2) and missing slices (req. 2) are supported. The user interface allows the setting of possible slice thicknesses to take the technical settings during cutting into account. The user can compare the length of the current stack of slices to the expected length (based on the tissue dimension) to detect missing slices (req. 2).

**Fig. 6:**
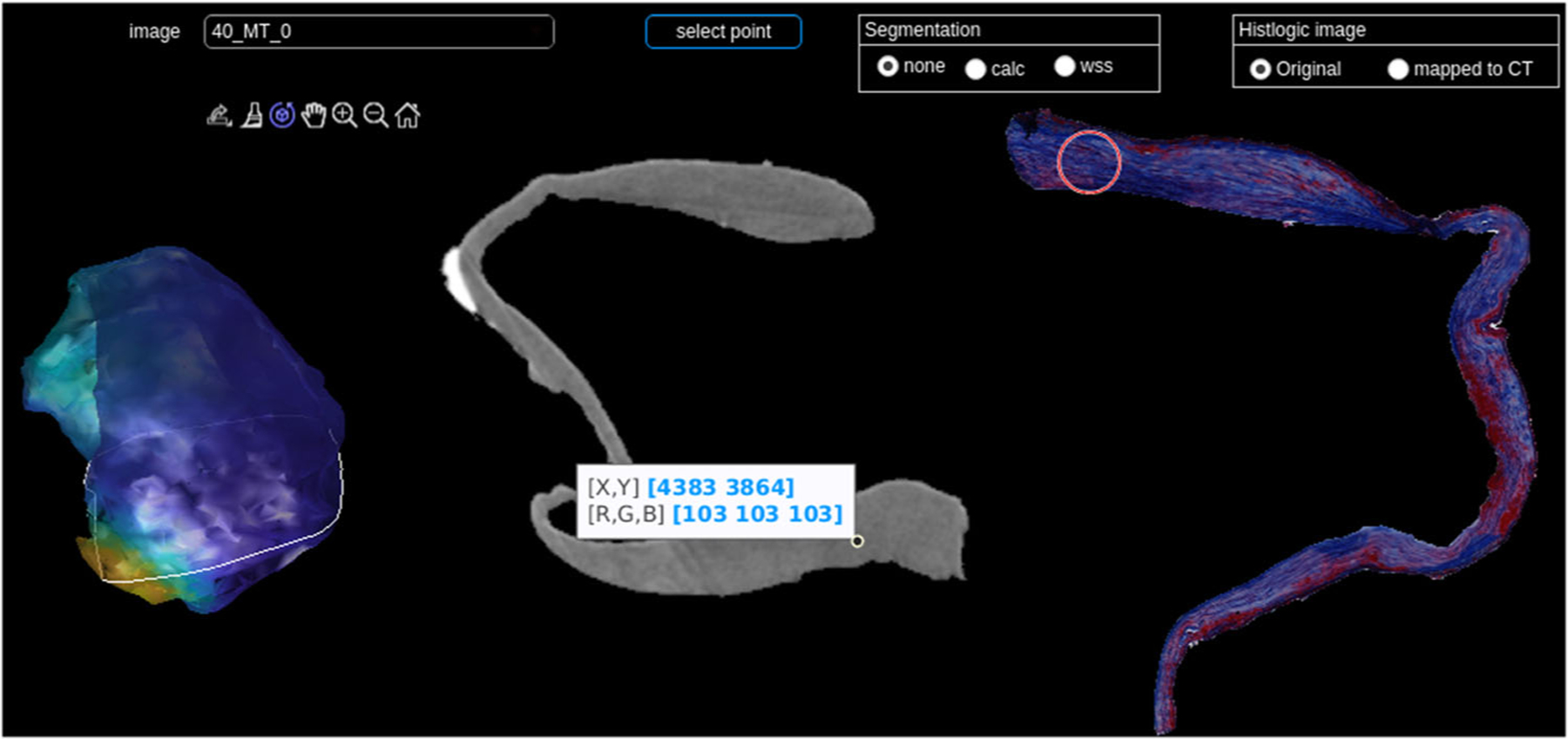
Visual exploration of combined micro-CT and histology; left: 3D model with WSS, the white line indicates the current slice; middle: segmented micro-CT image, right: segmented, corresponding MT-stained histologic image

The connection between micro-CT and histology can be interactively explored in the developed viewer, see Fig. 6. Selecting a point in the micro-CT image shows the corresponding area in the histologic image. The program shows the resected IA dome with WSS information on the left. The micro-CT image is displayed and information on calcification and WSS can be superimposed. On the right, the histologic image (either the segmented image or the one mapped to micro-CT, recall Fig. 5) is shown. When mapping the histologic data to micro-CT, small details are lost in contrast to major aspects, like dominant colors in MT-stained images. The blurring depends on the similarity of the shape of the histologic image and the micro-CT image.

We verified the mapping algorithm by transferring the histologic image to its own shape and the resulting image was very similar to the original one with a correlation coefficient of 0.989.

**Fig. 7:**
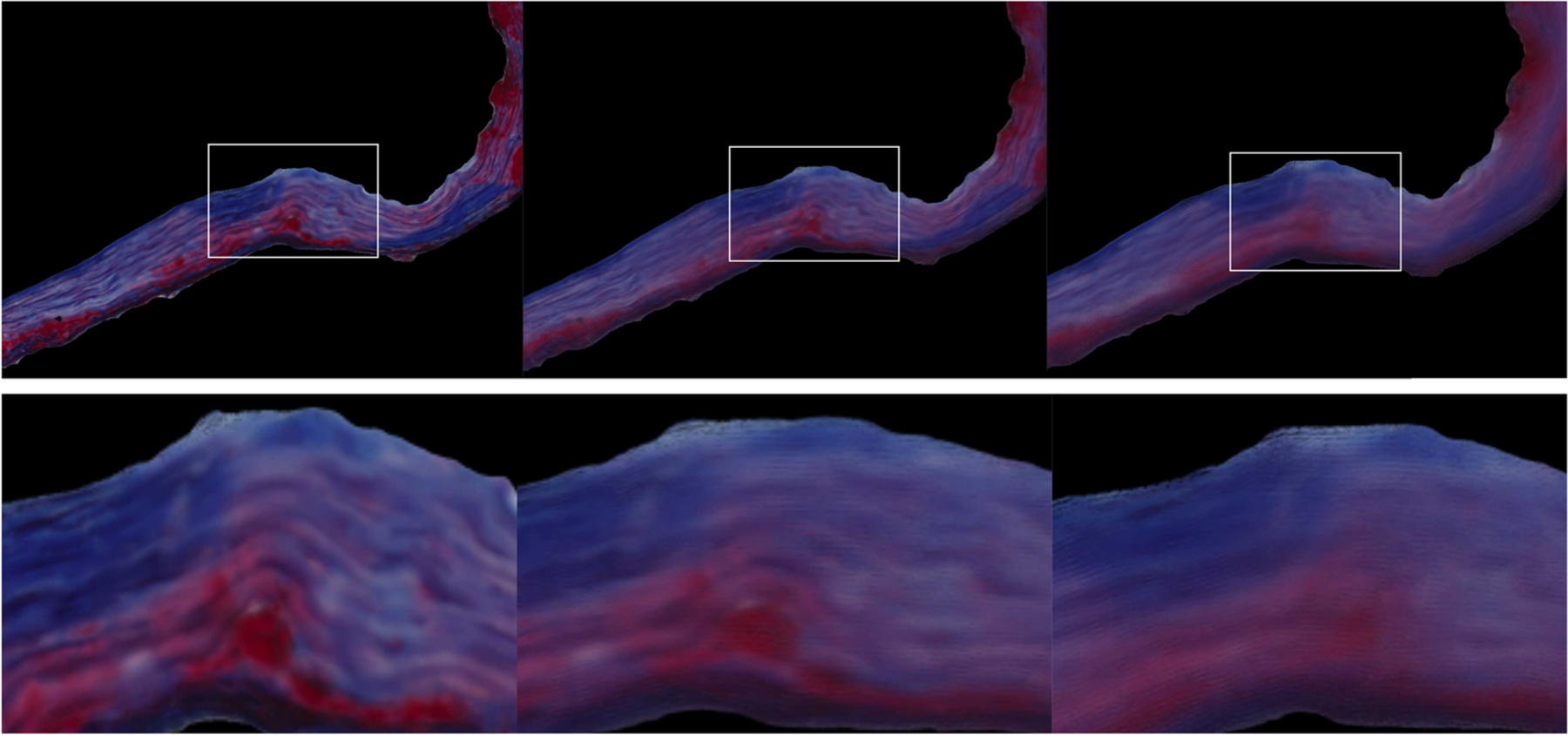
Detail view of mapping into own segmentation mask (left), slightly dilated mask (disk size 15, center) and more dilated mask (disk size 50, right). Masked regions are shown at the bottom

We systematically decreased the similarity between the histologic image and mask by applying a dilation with different-sized disks on the mask. The correlation coefficient decreased with increasing dilation (0.9496 for disk size 5, 0.9107 for disk size 15, 0.8412 for disk size 50). The results are shown in Fig. 7.

The virtual inflation transformed the tissue mesh to better fit the aneurysm, yielding a small change in the volume (4%). The mapping algorithm can be used for images with every staining (req. 1). Together with the virtual inflation, the mapping algorithm addresses the problem of deformation of the tissue (req. 3).

Finally, the visual exploration combines hemodynamically relevant parameters from preoperative data with image data of the tissue collected during surgery (req. 4).

### Informal evaluation

Two medical experts with experience in histologic image analysis examined the data based on the program using the think-aloud method. The resected dome only covered a small part of the IA. Compared with the rest of the aneurysm, the WSS was not conspicuous. Focusing on the WSS at the resected dome, the middle part of the tissue had a lower WSS than the outer parts. The experts stated that the transition from low WSS to high WSS is interesting, especially the possibility to compare this region with the different histological stainings. In the resected dome sample no correlation between calcification and histologic particularities was found. Calcification was mostly present in one half of the tissue. In this part, the WSS was slightly higher.

**Fig. 8:**
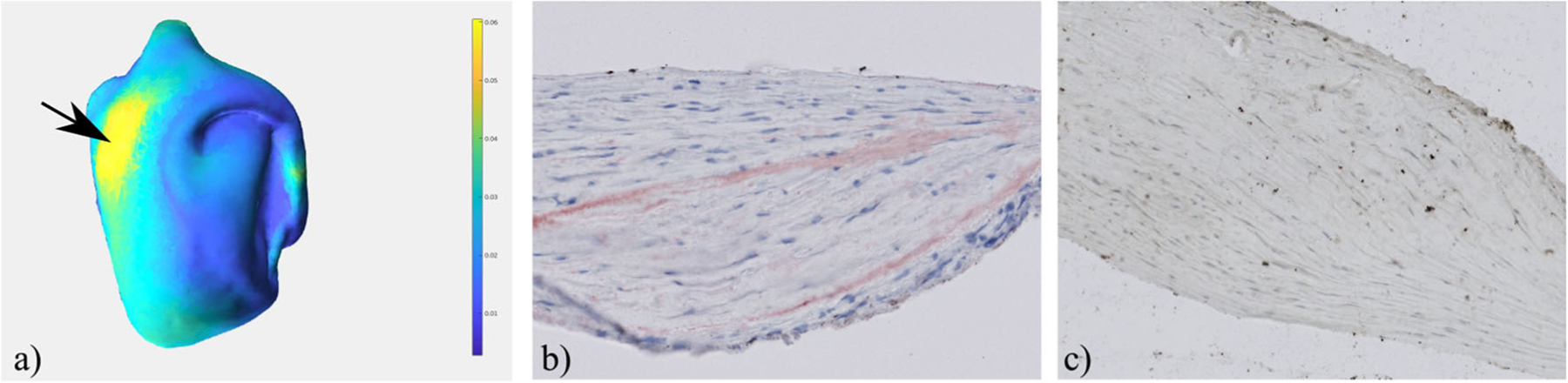
Exploration of the resected IA dome by mapping the wall thickness to color (**a**) and analyzing corresponding histological images. The ORO staining shows a lipid accumulation (red), especially in the region with increased wall thickness (**b**). The aSMA staining reveals a smooth muscle cell structure that is globally organized for this area (**c**). Also, a slight loss of smooth muscle cells was visible in the area of lipids, when comparing the adjacent sections

The experts also compared the thicker part of the resected tissue with the different histologic images provided by our visual exploration approach. First, they focused on the assessment of wall thickness and cellularity and analyzed the correlated HE and MT staining. In addition, they detected lipid accumulation in the ORO-stained images and a slight loss of smooth muscle cells in the aSMA-stained images, see Fig. 8. These findings are very important for understanding the composition of the IA wall.

## Discussion

The presented datasets are rarely available and with an increasing number of endovascular IA treatments, fewer surgical interventions are performed. This further limits the collection of resected dome tissue for research purposes, and histologic analysis becomes less available.

Although a strong deformation during the tissue processing and differences in histologic stainings prevent an automatic co-registration, histologic information is necessary for understanding aneurysm wall composition. The presented pipeline can combine the multimodal data and provides insight into the complex IA wall and associated hemodynamics even when an automatic registration is not possible.

For the presented approach, the length of the sample was approximated based on the sum of all section thicknesses and was used as a guide in the slide positioning tool. This must be used carefully, as it might be misleading due to differences in measurements in various modalities and tissue shrinkage during the fixation process [28,29].

Our pipeline can be applied to any IA, provided that the required multimodal imaging datasets are available. Since we focus on the aneurysm sac, separation of the aneurysm from the parent vessel should be performed, as carried out in previous work [21].

This study has several limitations. As mentioned above, the data sets are very rare, which justifies such a detailed analysis and demonstration of proof-of-concept. Unfortunately, research with a focus on the IA wall mainly explores tissue samples collected during surgical clipping, whereas the often small number of samples limits the explanatory power. Jabbarli et al. [30] compared the results of several studies for IA wall composition to extract a detailed pathophysiological wall model accounting for the number of patients and the existence of conflicting results during their evaluation. Subdivision of aneurysm wall types varies, e.g. five wall types (atherosclerotic, hyperplastic, thin, rupture site, and normal) based on surgical videos [14] or subdivision into thick, intermediate and super-thin translucent tissue based on histological and intraoperative observational studies [31]. This heterogeneity is also reflected in aneurysms with blebs. The evaluation of their wall composition yields walls of varying structures [22]. For giant aneurysms, the triple-layered microstructure of aneurysm walls as well as an intraluminal thrombus could be identified for most cases, again, the walls exhibited heterogeneous thicknesses [32].

In conclusion, to provide more generalizable results, a larger database is required. In addition, only a small part of the aneurysm wall can be collected during surgery and the tissue is deformed during the process. The preoperative 3D model only shows the aneurysm lumen and not the aneurysm wall tissue. This complicates the registration of the aneurysm to the collected tissue. Therefore, the accuracy of the WSS mapped to the micro-CT image is limited. The pitfall of various stainings of histologic samples is the missing ground truth information about the 3D shape. To cope with this limitation, our visual exploration pipeline guides the positioning of slides, see Fig. 3. The concept of our pipeline illustrates how multimodal data can be combined to fulfill the presented requirements. Since it is tailored to the presented data set, no general software tool is shareable right now.

In this study, no correlation between histology, calcification and WSS was evident. A further limitation is the small variance of the WSS at the collected tissue. Tissues from other areas or IAs might be more expressive, but such datasets are rarely available. Future work could aim at phantom studies, as carried out for validation of the virtual inflation [15]. However, different parts of the aneurysm wall exhibit different elastic properties [33] which might not be realized with a phantom.

## Conclusion

We presented a pipeline for visual exploration of multi-modal aneurysm data comprising histologic and micro-CT images as well as 3D surface meshes of the aneurysm and the resected dome. The proposed methods allow the combination of 2D histology with 3D micro-CT data, even if the histologic data is incomplete, comprises a variety of stainings or is insufficient for 3D reconstruction on its own. It can be used to find correlations between hemodynamic forces and wall characteristics like histologic structures, wall thickness or calcification.
